# Service Evaluation: Yoga Class Benefits for Mental and Physical Health in Mother and Baby Unit

**DOI:** 10.1192/bjo.2024.499

**Published:** 2024-08-01

**Authors:** Akvile Nikitina, Zeyn Green-Thompson

**Affiliations:** Norfolk and Suffolk Foundation Trust, Norwich, United Kingdom

## Abstract

**Aims:**

During the perinatal period women are at increased risk for mental health illness. It is estimated that around 0.5 in 1000 deliveries will result in admission to the mother and baby unit (MBU). Recovery is achieved by combining pharmacological treatment with holistic approaches. The majority of MBU settings will offer a variety of sessions that aid relaxation, reflection, and bonding. We have chosen to trial an additional service – Yoga class. It is known that Yoga is beneficial not only for strength, flexibility, and chronic pain but also for improved concentration, relaxation, and anxiety reduction.

**Methods:**

Service evaluation took place in 8 bed, inpatient MBU. Selection criteria included non-pregnant women who had 4–6 weeks postnatal health check, were interested in trialling the class and were willing to complete pre- and post-class selected questions from the Dialog scale. The total number of Yoga classes conducted was 9 but there was no set number of classes for patients to commit to. Sessions were run between October 2023 and February 2024. Dialog scale was selected as a well-established outcome measure within the ward. We measured 3 areas by a Dialog scale (physical health, mental health, and leisure). The rating range was 1–7 with 1 being totally dissatisfied and 7 totally satisfied. Questions were completed before and after the class.

**Results:**

In total 7 patients attended at least 1 Yoga class. We have calculated pre- and post-class average scores to measure change in selected outcomes. Physical health self-reported evaluation improved from 4.09 (SD = 0.79) to 4.48 (SD = 0.71). Mental health score improved from 3.61 (SD = 0.96) to 4.29 (SD = 0.99). Leisure score rose from 3.67 (SD = 1.3) to 4.34 (SD = 0.55). From the class record it was noted that overall, the uptake of the class was encouraging with 85% of patients returning to the Yoga with on average completion of 3 classes. 6 out of 7 patients did not attend further classes due to discharge or other commitments rather than withdrawing from classes.

**Conclusion:**

From the collected data we can see that Yoga classes appear to be associated with moderate improvements in mothers’ mental and physical health, at least immediately post-class. Whether this translates into long-term benefits remains unknown. Our service evaluation indicates that Yoga can be a beneficial part of holistic management for mothers in the MBU setting. In the future, this study could also involve pregnant mothers, who are an important population within the MBU setting.

